# Rising cases of meningococcal disease in Florida yet again: an urgent concern

**DOI:** 10.1097/JS9.0000000000000198

**Published:** 2023-02-16

**Authors:** Aroma Naeem, Shehroze Tabassum, Abubakar Nazir, Maleeka Z. Khan, A. Awuah Wireko

**Affiliations:** a King Edward Medical University, Lahore, Pakistan; b Sumy State University, Sumy, Ukraine

*Dear Editor*,

One of the most serious and deadly conditions is bacteremia, which can consequently result in septic shock due to endotoxemia. *Neisseria meningitides* causes meningococcal infection, manifested as meningitis or septicemia or sometimes both. Initial step of *Neisseria* infection is colonization of pharynx and is usually asymptomatic. The natural reservoir of this bacterium is humans. It reaches the meninges from the nasopharynx, crossing the mucosa of the nasopharynx and traveling along the perineural sheath of the olfactory nerve through the cribriform plate, resulting in meningitis. Signs and symptoms of the disease can range from a flu like illness to complicated meningococcemia. It can initially present as sudden onset fever, headache, nausea, vomiting or diarrhea, severe aches, a dark purple rash, and sore throat. Worsening symptoms include neck stiffness, photophobia, and a hemorrhagic rash. Route of transmission includes respiratory droplets and direct close contact with the person suffering from meningococcal disease. Children younger than 5 years are not immune against the antigens of *N. meningitides* due to the presence of its polysaccharide capsule. Risk factors associated with this infectious disease include, lack of good hygiene, immunologic susceptibility, lack of awareness, and repeated oral contact with objects in the surroundings. Child care centers are specifically associated with this infection. Two age groups are more susceptible to invasive meningitis caused by *N. meningitis.* These include infants lacking maternal antibodies and adults, in whom nasopharynx colonization of this bacteria is quite high^1^,^2^. The virulence factors of *N*. *meningitis* include the polysaccharide capsule and pili^1^. The capsule increases the invasion of bacteria by inhibiting the process of phagocytosis and increases the chances of bacterial survival during invasion in the bloodstream and central nervous system. Pili is involved in the attachment and colonization of organisms. Cassette mechanism is the reason for pili’s antigenic variation and enables the bacteria to run off the immune system of the host. The capsular polysaccharides are antigenic and form the basis of serogroups^2^. There are 12 serogroups, and these serogroups include A, B, C, H, I, K, L, X, Y, Z, W-135, and 29F. The most common serogroups involved in causing meningococcal disease are A, B, C, W-135, X, and Y.

Invasive meningococcal disease (IMD) can exist as an endemic disease or as an epidemic. IMD incidence occurs mainly in infants under 1 year of age, teenagers, and young adults. Thus, these groups are highly susceptible to IMD. The main factor associated with increased incidence in infants is a deficiency of bactericidal antibodies after the levels of maternal antibodies fall. However, peak incidence in adults is due to an increased rate of nasopharyngeal colonization^3^. IMD can also affect older patients during severe outbreaks^3^. Patients with complement system deficiency and asplenia are also at high risk of developing IMD. In 2019, 23 cases of meningococcal disease were reported in Florida, which dropped to 17 cases in 2020 and again rose to 27 cases in 2021^4^. Up until June 2022, 26 cases and seven deaths had been recorded because of meningococcal disease in Florida. Twenty-four cases and six deaths were documented in bisexuals and gays, and it is considered one of the worst meningococcal disease outbreaks in gays and bisexual^5^. As of August 2022, 48 cases had been recorded in Florida^4^. The trend of meningococcal disease cases in Florida has been shown in Figure 1 4. A low incidence of endemic IMD ranging from 0.3 to above 3.0 cases per 100 000 population is found in developed regions with most cases due to C and B serogroups^6^,^7^. The Active Bacterial Core Surveillance Network targets about 13% of the total population of the United States. An annual incidence rate of 0.55 cases per 100 000 people was recorded from 1998 until 2007, but a drop of 64.1% in the annual incidence of meningococcal disease has been noticed since 2007 in the United States. Infants below 1 year old are a highly affected group, with a disease incidence rate of 5.38 per 100 000 infants. Children from 1 to 4 years old in the United States have a disease incidence of 1.4 per 100 000 children, and adults have a disease incidence of 0.78 per 100 000 adults. Overall, 39% of isolates were serogroup B, followed by Y (30%) and C (25%). Outbreaks of serogroup B disease have resulted in IMD in college students in the United States that have captured the publics’ attention. Other risk factors associated with IMD include more than one kissing partner or bar attendance. The US Food and Drug Administration has permitted the use of meningococcal serogroup B vaccine for undergraduates through an application called an Investigational New Drug Application after a response from the Center for Disease Control and Prevention (CDC) regarding the high incidence of IMD. Mortality rates range between 5 and 15% for treated patients and about 50% for untreated patients, even after advancements in the prevention of disease^8^.

**Figure 1:**
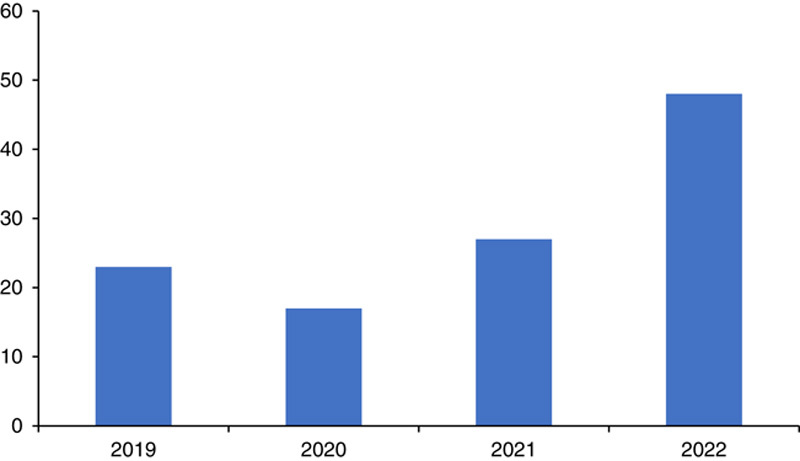
Meningococcal disease cases in Florida.

The immune system is very important in defending the host against meningococcal disease. Major risk factors of meningococcal disease are those diseases which are associated with immunocompromised conditions like HIV infection, inherited deficiency of the complement system, splenic dysfunction, and acquired complement deficiency^9^. Groups at highest risk of infection are those who are living in closely populated spaces like military recruits. There are greater incidences in black people than in white people. The exposure, both active and passive, to tobacco smoke (odds ratio: 1.8; 95% CI: 1.0–3.3; *P*=0.05) is another significant risk factor. The risk of contracting this disease is higher in men than women (1.3 : 1). However, the mortality rate is much higher in women than in men^9^.

The world has already been through the whirlpool of several bacterial and viral outbreaks^10^–^12^ in this year, so there is a dire need for strict measures for public safety. Outbreak serogroup specific vaccination is recommended for any meningococcal outbreak. In response to the ongoing outbreak, the CDC has recommended the MenACWY vaccination for gay or bisexual men living in Florida and those traveling to Florida. In addition to these, people at increased risk of meningococcal disease are encouraged to get their routinely recommended two-dose primary series of vaccines, followed by a booster every 5 years. These high-risk people include those with HIV, functional or anatomic asplenia, persons receiving a complement inhibitor, and microbiologists exposed to the isolates of *N. meningitides*. Protective antibody levels are achieved 2 weeks following vaccination. For people vaccinated at age less than 7 years, first booster dose is recommended 3 years after primary doses and then after every 5 years. For people who received their primary doses after the age of 7 years, a booster dose every 5 years is recommended^13^,^14^. These vaccination doses are covered by health insurance and are also available for free at county health departments^13^.

Expanded antimicrobial chemoprophylaxis in conjunction with vaccination is used for closed contacts in organization-based outbreaks. This is important for the prevention of secondary outbreaks. Close contacts refer to a proximity of less than 3 feet for more than 8 hours. Several antibiotics used for chemoprophylaxis are ceftriaxone, rifampin, and ciprofloxacin. Due to excellent efficacy, it is recommended to start prophylaxis without waiting for laboratory test results in the case of strong suspicion^15^. Since vaccine takes 2 weeks to provide protective antibody, antibiotics in close contacts help in prevention of contracting disease in this period. However, vaccination must be administered to all susceptible individuals.

The general population should be educated about signs and symptoms and the promotion of early care-seeking behavior. Both print and electronic media must be engaged in spreading awareness among the public. There is no recommendation of travel restrictions or ban on social gatherings in the outbreak as they have no effect on outbreak course. Promotion of vaccination, especially in the vulnerable population, is the key step in the prevention and control of outbreaks. Meningococcal disease outbreaks are dynamic. Re-evaluation to determine the status of disease will be needed to implement public health measures accordingly. In community-based outbreaks such as the one Florida is facing, the CDC recommends re-evaluation 1 year after the last reported case of the outbreak. We believe if these measures are taken effectively, Florida can be meningococcemia-free region.

## Ethical approval

Not applicable.

## Consent

Not applicable.

## Source of funding

None.

## Authors’ contributions

A.N.: conceptualization. A.N., S.T., A.N., M.Z.K., and W.A.A.: writing. A.N. and S.T.: review with critical comments and editing.

## Conflicts of interest disclosure

The authors declare that they have no financial conflict of interest with regard to the content of this report.

## Research registration unique identifying number (UIN)

None.

## Guarantor

All authors.

## Provenance and peer review

Not commissioned, externally peer reviewed
